# Evaluation of the Mechanical Properties of Different Dental Resin-Based Materials After Submersion in Acidic Beverages

**DOI:** 10.3390/dj13010004

**Published:** 2024-12-25

**Authors:** Răzvan Constantin Brânzan, Ionuț Tărăboanță, Cristina Angela Ghiorghe, Simona Stoleriu, Vlad Cârlescu, Andra Claudia Tărăboanță-Gamen, Sorin Andrian

**Affiliations:** 1 Faculty of Dental Medicine, Grigore T. Popa University of Medicine and Pharmacy Iasi, Str. Universitatii No. 16, 700115 Iasi, Romania; 2 Department of Mechanical Engineering, Mechatronics and Robotics, Gh. Asachi Technical University, 67 Dimitrie Mangeron Str., 700050 Iasi, Romania

**Keywords:** resin-based materials, scratch resistance test, Vickers microhardness, Young’s modulus, tribology

## Abstract

**Background**: The aim of this study was to evaluate the influence of acidic beverages on the mechanical properties of various dental resin-based materials. **Methods**: A total number of 160 samples were prepared using four types of resin-based materials—Group A (*n* = 40): flowable composite, Group B (*n* = 40): heavy-flow composite, Group C (*n* = 40): resin-based sealant and Group D (*n* = 40): nano-hybrid composite. Then, the samples were distributed into four subgroups according to the submersion solution: *a* (*n* = 10): artificial saliva, *b* (*n* = 10): coffee, *c* (*n* = 10): cola and *d* (*n* = 10): red wine. The Vickers microhardness, Young’s modulus of elasticity and scratch resistance were assessed using a CETR UMT-2 tribometer. **Results**: The obtained results showed that 14-day submersion of the resin-based materials in coffee, cola and red wine solutions significantly (*p* < 0.05) decreased the microhardness values (VHN), Young’s modulus of elasticity and scratch resistance. Fourteen days of storage in coffee decreased the microhardness values of flow resin from 117.5 to 81.59 VHN (*p* < 0.001) whereas the values of the nanohybrid resin decreased from 125.5 to 89.4 (*p* < 0.001). The elasticity modulus of the heavy flow resin showed a decline from 15.57 to 10.50 GPa after 14 days’ submersion in coffee (*p* < 0.001), and from 21.29 to 13.10 GPa for the nanohybrid resin after immersion in cola (*p* < 0.001). For the scratch test, the resin-based sealant showed a significant decrease after 14 days of storage in coffee, from 0.34 to 0.02 units. **Conclusions**: The submersion of conventional nanohybrid, flowable, heavy-flow composite resins and resin-based sealants in coffee, cola and red wine solutions changes the mechanical properties (Young’s modulus of elasticity, Vickers microhardness and scratch resistance). The most resistant resin-based material to acid attack was the conventional nanohybrid composite resin, followed by heavy flow resin, flowable resin and resin-based sealant.

## 1. Introduction

The use of composite resins as direct restorative materials has become widespread in recent decades as patient interest in esthetic restorations has increased. Composite resins are preferred by clinicians due to their minimally invasive preparations, outstanding esthetic properties, adhesion to dental tissues and enhanced durability [1].

Over the years, the composite materials used in dentistry have been improved, showing now enhanced physical, chemical and esthetic properties, which allow them to be used in different clinical situation [2]. Thus, due to the contemporary lifestyle, the consumption of carbonated drinks, coffee, black tea and alcoholic beverages has grown significantly, posing potential risks to resin-based restorative materials. The acidic nature of these beverages, characterized by their low pH, may compromise the material’s structure, alter filler properties and weaken the bond between fillers and the organic matrix, ultimately impacting the longevity of dental restorations [3]. The decision made by the clinician regarding the selection of the most appropriate composite resin depends also on the material structure and the resistance to chemical and mechanical wear [1,3]. In the oral cavity, the restorations are constantly subjected to changes induced by the oral environment, either by exposure to acidic foods or beverages or by the occlusal masticatory forces or the excessive forces that may occur in some circumstances [4].

The wear resistance of direct restorative materials is still an open issue for clinicians, patients and dental materials manufacturers [5]. Some studies show that the wear resistance of composite resins depends mainly on the size, the distribution, the mechanical properties and the content of inorganic filler particles [2,6]. Different strategies have been proposed in order to improve the resistance to abrasion of resin-based restorative materials, like lowering the size of inorganic particles up to the nanometer scale, the mixture in the same composite material having different filler particle sizes, or the addition of inorganic particles with increased hardness to a lower hardness matrix [3]. Generally, the wear behavior of dental composite resins is influenced by the morphology, the particle uniform distribution and the concentration of inorganic fillers [7].

Restorative materials can be degraded by factors having intrinsic origin, such as gastric acid, or factors with extrinsic origin, like constantly consumed acidic foods or beverages [8]. The pH may cause material degradation by hydrolysis and damage of the polymer matrix. In the case of alcoholic beverages, it has been observed that they cause the plasticization of the polymer matrix [9]. As for red wine, it has been reported to cause erosive wear of resin-based restorative materials and to increase surface roughness [9,10]. For composite resins, knowledge of their mechanical and tribological properties as well as their behavior in different clinical situations is essential for choosing the most optimal material in different clinical situations. To test these properties, nanoindentation studies and tribological investigations, such as determination of the modulus of elasticity, microhardness and scratch resistance, have proven to be very effective [11].

The aim of this in vitro study was to evaluate the influence of immersion in different acidic beverages on the mechanical properties (Vickers microhardness, Young’s modulus of elasticity and scratch resistance) of different resin-based direct restorative materials; a nano-hybrid composite resin, a flow composite resin, a heavy flow composite resin and a resin-based sealant were evaluated. The null hypothesis was that different periods of immersion in acidic beverages have no effect on the tribological properties of resin-based materials.

## 2. Materials and Methods

The study was conducted in accordance with the Declaration of Helsinki, and approved by the Ethics Committee of “Grigore T. Popa” Unversity of Medicine and Pharmacy of Iași, Romania (no. 291/10 April 2023).

A flowable composite resin (GrandioSO Flow, Voco, Cuxaven, Germany), a heavy flow composite resin (GrandioSO Heavy Flow, Voco, Cuxaven, Germany), a resin-based sealant (GrandioSeal, Voco, Cuxaven, Germany) and a nanohybric composite resin (GrandioSO, Voco, Cuxaven, Germany) were included in this study. Red wine, coffee and a coca-type beverage (Pepsi Cola) were chosen for acidic immersion of the materials for different periods of time: 1, 7 and 14 days. Details on the manufacturer and composition of the materials used in this study are presented in Table 1.

Sample calculation was performed using G*Power software version 3.1 (Heinrich-Heine Universitat, Düsseldorf, Germany) with an effect size f of 0.30 considered to be a small effect according to Cohen’s classification, an alpha value of 0.05 and a statistical power of 0.8. The obtained results indicated a minimum of 128 samples for the study groups, or 32 samples per group.

**Figure 1:**
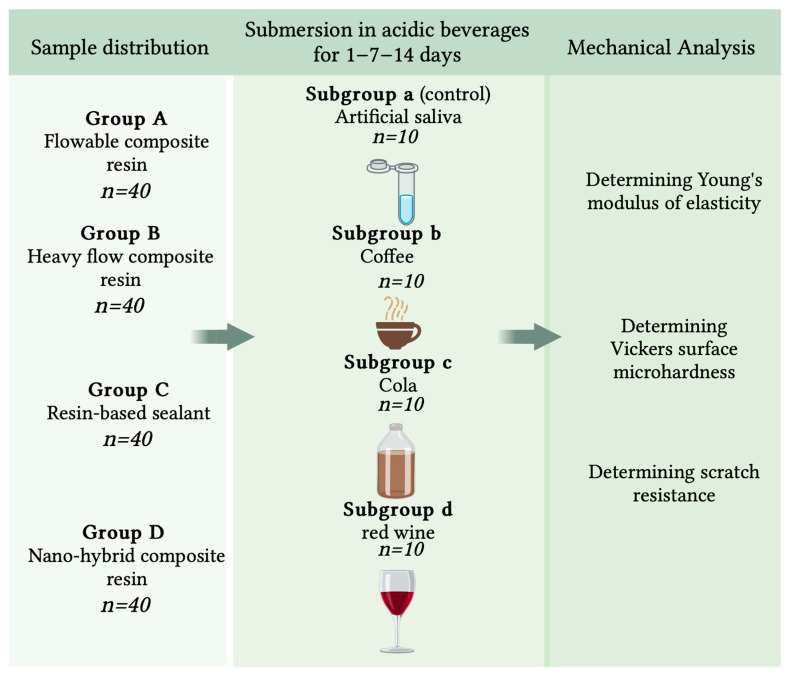
Study design (Created in BioRender. Taraboanta, I. (2024) https://BioRender.com/r74p626) accessed on 5 July 2024.

A total number of 160 samples (including 32 samples in control group) were prepared in a rectangular shape (25.0 ± 0.1 mm long, 10.0 ± 0.1 mm wide and 3.0 ± 0.1 mm thick) using a customized acrylic mold. The distribution of the samples into groups and the study design are presented in Figure 1.

After placing the materials in the mold, they were covered with a cellulose acetate strip and a glass slide over which a 0.5 kg weight was applied for 20 s to ensure uniform material distribution and to prevent air bubble formation. Subsequently, through the thickness of the celluloid matrix, the material was light-cured using the Elipar DeepCure-S; LED Curing Light (3M ESPE, St. Paul, MN, USA) with a wave length of 430–480 nm and a light intensity of 1.470 mW/cm^2^ (−10%/+20%). The accuracy of the curing light lamp was assessed using a radiometer Bluephase Meter II (Ivoclar Vivadent, Schaan, Lichtenstein). The materials were polymerized for 20 s according to manufacturer recommendations. The samples were then finished with Sof-LexTM Spiral Finishing Wheels Refill abrasive discs made of thermoplastic elastomer (3M ESPE, St. Paul, MN, USA) using a conventional speed of 20,000 revolutions per minute. Initially, the finishing procedure was performed using the beige disk, designed for removing scratches, smoothing and finishing with the highest grit size for 30 s, and, subsequently, the white disk designed for final polishing was used for the polishing procedure for 30 s [12]. A single operator was involved in the sample preparation process. The research methodology is shown in Figure 1.

The study samples were divided into 4 study groups according to the used material, and the distribution of the samples by groups is shown in both Figure 1 and Table 1. The samples within each group were further equally divided into 4 study subgroups according to the submersion solutions: subgroup a—AFNOR-control artificial saliva (Biochemazone™, Leduc, AB, Canada) (*n* = 10); subgroup b—soluble coffee (Nescafe Brasero, Nestle, Vevey, Switzerland) (*n* = 10); subgroup c—cola-type beverage (Pepsi cola original, PepsiCo, Harrison, HY, USA) (*n* = 10); and subgroup d—dry red wine (*n* = 10). The soaking solutions were changed every day. The submersion of samples was performed in an incubator (Biobase BJPXH30II, Biodusty, Jinan, Shandong, China) at a constant temperature of 37 °C for 14 days. Tribological measurements were made immediately after sample preparation and after 1, 7 and 14 days of submersion, respectively. Each time the samples were removed from the acidic solution they were washed with distilled water and dried. The pH of the submersion solution was checked each time the solution was changed using a portable pH meter (Thermo Scientific Eutech pH 5+, Thermo Scientific Eutech pH 5+, Vernon Hills, IL, USA). The mean pH value for each solution was of 7.4 for artificial saliva (control group); 5.33 for arabica coffee; 2.71 for Pepsi-Cola carbonated soft drink and 3.85 for red wine.

### 2.1. Determination of Mechanical Properties

Measurement of Vickers microhardness and Young’s modulus of elasticity was carried out using a Tribometer CETR UMT-2, Version 1.01 software (Bruker Corporation, Berlin, Germany). The device assesses the material microhardness using the indentation procedure with a square-based pyramid with a 136° angle tip. The microhardness is automatically determined by the equipment’s software based on the values of loading force and penetration depth and not by measuring the diagonals of the indentation marks formed in the material after penetration of the indenter as is the traditional method. The loading force was 1 kgf = 9.8 N for 15 s, and the obtained values were reported in terms of Vickers Hardness Number (VHN). The pressing force was applied using a force sensor attached to the tribometer, and the penetration depth was determined using a 0.05–250 μm capacitive sensor. The pressing force Fz and penetration were recorded during the whole indentation procedure. The procedure stages were as follows: reload with 10% of maximum force(1 N for 5 s), microindentation, loading: with increasing force over time: from 1 N to 10 N for 30 s, constant force (hold at maximum force of 10 N for 15 s), unloading: (decreasing force over time: from 10 N to 1 N for 30 s), constant force (hold at 1 N force for 15 s) and total discharge (from 1 N to 0.1 N for 5 s) [13].

The samples were fixed on the linear table of the tribometer, and the scratch test was carried out using a 0.4 mm tip radius blade fixed on a spring adapter in the force sensor. The scratch test was performed at a time-varying force ranging from 0.1 to 10 N over a distance of 10 mm at a speed of 0.167 mm/s.

The scratch resistance was evaluated by analyzing the variation graphs of the coefficient of friction (COF) with different forces of 1, 5 and 10 N, obtained at the end of the 14 days of submersion in the tested solutions [14].

For each sample, the Vickers microhardness, Young’s modulus of elasticity and scratch resistance were tested 3 times, with the final value being determined as the mean of the 3 measurements.

### 2.2. Statistical Analysis

Statistical analysis was performed using IBM SPSS 29.0.0 software. The normality of the distribution of the data was checked using the Shapiro–Wilk test, while the homogeneity of variances was tested using Levene’s test. The T-test and One-way ANOVA and Tukey post-hoc parametric statistical tests were used for the comparative analysis of the data obtained from the Vickers microhardness and Young’s modulus of elasticity tests, and the Kruskal–Wallis non-parametric test was used for the analysis of the data resulting from the scratch resistance analysis. For all statistical tests, the significance threshold value was set at 0.05.

## 3. Results

### 3.1. Microhardness Evaluation Results

**Figure 2:**
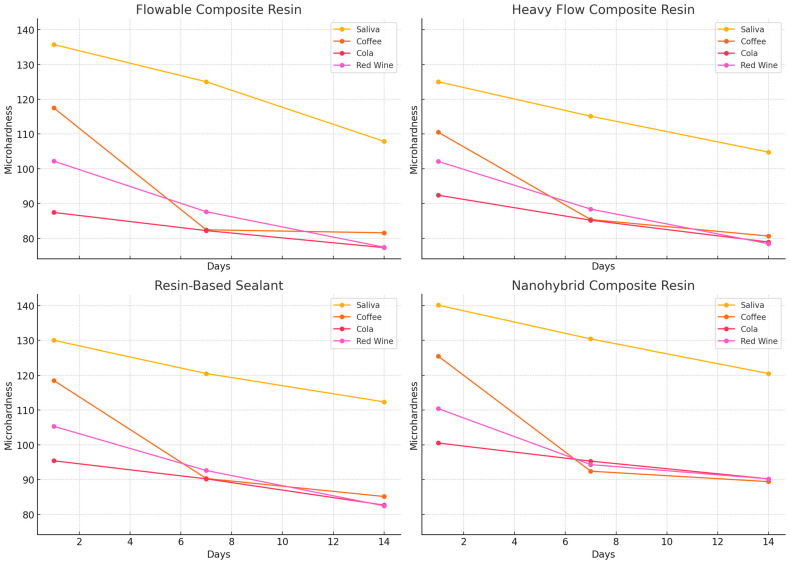
The evolution of microhardness VHN values of each tested dental material after submersion in each solution for 1, 7 and 14 days. Each line represents the performance of a material in a specific solution, with markers showing the points of measurement at each time interval.

In Figure 2 is presented the evolution of the microhardness VHN values of each group after submersion in each tested beverage for 1, 7 and 14 days.

Based on the data presenting the evolution of the microhardness values of the tested materials at 1, 7 and 14 days of evaluation after submersion in the 4 tested solutions, it can be observed that for all materials the highest variations of the mean VHN values were recorded after submersion in the coffee solution. These values were as follows: for samples made of flowable composite resin (Group A), the values decreased from 117.50 ± 7.82 on day 1 to 81.59 ± 8.75 on day 14. As for the heavy flow composite resin (Group B), the microhardness values decreased from 110.50 ± 7.82 on day 1 to 80.65 ± 21.26 on day 14. A similar evolution was also recorded for the resin-based sealant samples (Group C), with a variation of 33.38 VHN units, with the values decreasing from 118.50 ± 0.59 on day 1 to 85.12 ± 8.74 on day 14. Regarding the nanohybrid composite (Group D), it can be stated that the mean VHN values decreased from 125.50 ± 0.59 on day 1 to 89.40 ± 8.74 on day 14.

The statistical differences between the obtained values were also evaluated, so that in Group A, differences between the samples submersed in coffee solution for 1 day vs. 14 days (*p* < 0.001) and for 7 days vs. 14 days (*p* = 0.006) were obtained. For the samples in Group A submersed in red wine solution, significant differences were obtained between the values in day 1 vs. day 7 (*p* = 0.047); day 1 vs. 14 (*p* < 0.001); and day 7 vs. 14 (*p* = 0.012). Comparative assessment of differences between the control (saliva) and study sample values showed differences in day 1 between control vs. coffee/vs. cola/vs. red wine; for all these comparisons the level of significance was *p* < 0.001. On testing day 7, significant differences were recorded between the control vs. wine (*p* = 0.002), and as for test day 14, significant differences were found between the control and cola solution, respectively, and red wine, both with *p* values of <0.001.

The samples in group B showed differences between the values obtained after submersion in coffee solution for 1 day vs. 14 days (*p* < 0.001), and for 7 days vs. 14 days (*p* = 0.004). The differences between the values in the control group and those of the samples submersed in the tested solutions were also analyzed so that after 14 days of submersion, significant differences were found between the control samples and the samples submersed in cola solution and coffee, with a significance of *p* < 0.001.

In group C, significant differences were found between the samples submersed in coffee solution for 1 day vs. 7 days (*p* = 0.004) and for 1 day vs. 14 days (*p* = 0.009); and in red wine solution for 1 day vs. 7 days (*p* = 0.006) and for 1 day vs. 14 days (*p* < 0.001). It was also remarked upon that on testing day 1, differences were found between the control samples and those submersed in cola solution (*p* = 0.008) and red wine (*p* = 0.002), respectively. On testing day 7, differences were recorded between the control group and the samples soaked in cola solution (*p* = 0.007). For testing day 14, differences were observed between the control samples and those submersed in cola solution (*p* = 0.039) and red wine (*p* = 0.046).

In group D, significant differences were found between samples submersed in coffee for 1 day vs. 14 days (*p* < 0.001), in cola for 1 day vs. 14 days (*p* < 0.001) and in red wine for 1 day vs. 14 days (*p* < 0.001). It was also observed that after 14 days, significant differences were obtained between the control group and samples submersed in coffee (*p* = 0.027), in cola solution (*p* < 0.001) and in red wine (*p* < 0.001).

### 3.2. Young’s Elasticity Modulus Evaluation Results

**Figure 3:**
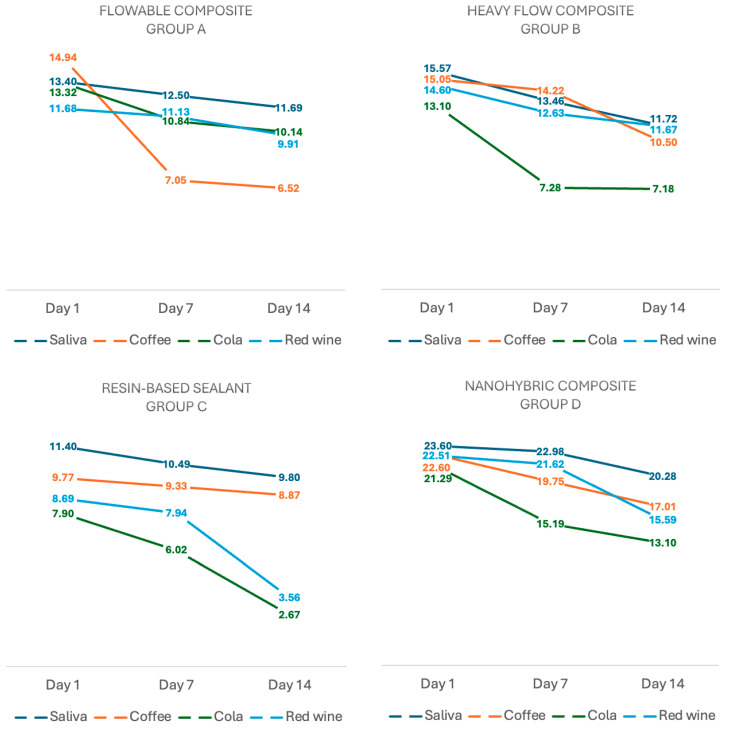
The evolution of Young’s elasticity modulus values (GPa) of each tested dental material after submersion in each solution for 1, 7 and 14 days. Each line represents the performance of a material in a specific solution, with mean values at each time interval.

As for the flowable composite resin, the highest value was recorded by the samples submersed in coffee solution on test day 1 (14.94 ± 0.59). Over time, all the values showed a decreasing trend, with the most significant variation being observed in the samples submersed in coffee solution, which on day 14 showed a significant reduction to a value of 6.52 ± 1.08, representing a total drop of 8.42 units. Heavy-flow resin samples also showed a downward trend of values, with the most significant variation recorded by the samples submersed in coffee solution, which on day 1 recorded a value of 15.57 ± 0.59 decreasing to 10.50 ± 1.65 on day 14 (Figure 3).

The resin-based sealants showed a downward trend in Young’s modulus of elasticity values after submersion in each of the four beverages. However, the samples soaked in cola solution stood out due to the values varying from 7.90 ± 0.17 on test day 1 to 2.67 ± 0.27 on day 14. For the same pattern with the previous materials, the nanohybrid composite resin values showed a downward direction from testing day 1 to day 14. The most significant variation in values was shown by the samples submersed in cola, decreasing by 8.19 units from 21.29 ± 1.15 on day 1 to 13.10 ± 0.64 on day 14.

In group A, significant differences were obtained between the samples submersed in artificial saliva for 1 day vs. 14 days (*p* < 0.001) and for 1 day vs. 7 days (*p* < 0.001), respectively. For the samples in group A submersed in cola, significant differences were obtained between the values of day 1 vs. day 7 (*p* = 0.002) and day 1 vs. 14 (*p* < 0.001), and the samples submersed in wine showed significant differences between day 1 vs. 7 (*p* = 0.008) and day 1 vs. 14 (*p* < 0.001). Comparative evaluation of the differences between the values of the control and study samples showed differences on day 1 between the control vs. coffee, vs. cola and vs. wine; for all these comparisons the significance level was *p* < 0.001. At the end of day 7 there were significant differences between the control vs. cola (*p* = 0.016) and vs. wine (*p* = 0.009), and at the end of day 14 of evaluation, significant differences were found between the control vs. cola and wine solutions; for both, the *p*-value was <0.001.

The samples in group B showed differences between the values obtained after submersion in coffee solution for 1 day vs. 14 days (*p* < 0.001) and 1 day vs. 7 days (*p* = 0.007); for the cola solution between day 1 vs. 7 (*p* < 0.001) and day 1 vs. 14 (*p* = 0.016); and for the samples submersed in wine between day 1 vs. 7 (*p* < 0.001) and day 1 vs. 14 (*p* = 0.024). Furthermore, the differences between the values of the control group and those of the samples submersed in the tested solutions were analyzed, and the results showed no statistical differences.

Within group C, there were significant differences between the samples submersed in coffee and cola solution, between the values reported after 1 day vs. 7 days and 1 day vs. 14 days, respectively, and for all these situations the *p*-values were <0.001. For samples submersed in red wine, there were significant differences between the values recorded at the end of day 1 vs. 14 (*p* = 0.002). In addition, it was noticed that by the end of day 7 of testing, significant differences were found between the control samples and those submersed in coffee (*p* = 0.004).

In group D, significant differences were found between the samples submersed in cola solution for 1 day vs. 7 days and for 1 day vs. 14 days (*p* < 0.001), respectively, and in wine for 1 day vs. 14 days (*p* < 0.001). It can also be mentioned that after 14 days of soaking in the three tested solutions, significant differences were obtained between the control group and the samples submersed in these solutions; for each of them a *p* value of *p* < 0.001 was obtained.

### 3.3. Scratch Resistance Testing Results

**Figure 4:**
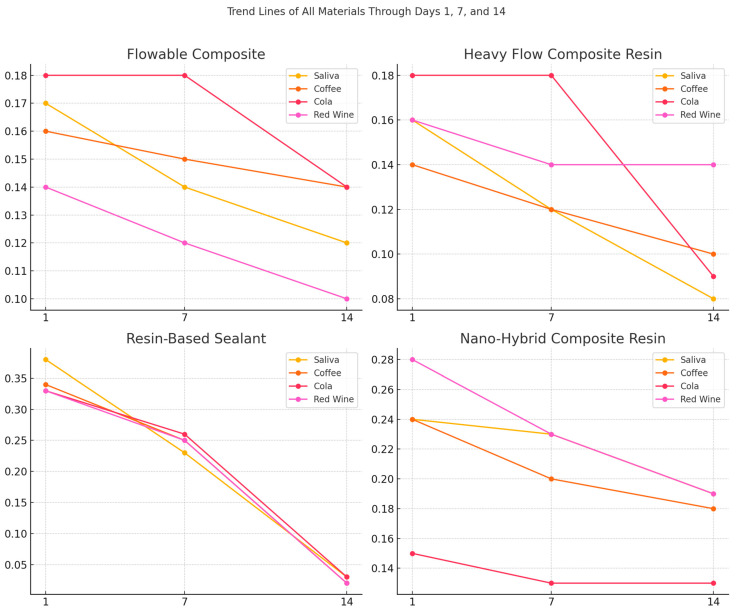
Trend lines representing the mean scratch resistance values of each tested material exposed to each acidic beverage over time (days 1, 7 and 14).

For the flowable composite resin, the greatest variation was observed after submersion in red wine, wherein the mean value decreased from 0.14 ± 0.04 on day 1 to 0.10 ± 0.04 on day 14, resulting in a decrease of 0.04 units (Figure 4).

As for the heavy-flow composite resin, the mean value decreased after submersion in the red wine solution from 0.16 ± 0.04 on day 1 to 0.14 ± 0.06 on day 14, suggesting a decrease of 0.02 units.

The resin-based sealants showed a large decrease after submersion in the coffee solution from 0.34 ± 0.15 on day 1 to 0.02 ± 0.01 on day 14, indicating a difference of 0.32 units. Regarding nanohybrid composite resins, it can be stated that submersion in red wine solution produced the most considerable decrease in mean values from 0.28 ± 0.11 on day 1 to 0.19 ± 0.18 on day 14.

Statistical analysis showed that in group A, there were significant differences between the values obtained after applying a force of 1 N vs. 5 N (*p* = 0.035) and 1 N vs. 10 N (*p* < 0.001) on samples submersed in artificial saliva. For samples submersed in coffee, differences were observed between values recorded after applying a force of 1 N vs. 5 N (*p* = 0.009) and 1 N vs. 10 N (*p* < 0.001). For samples submersed in wine, differences were found between values recorded after applying a force of 1 N vs. 5 N (*p* = 0.012) and 1 N vs. 10 N (*p* = 0.001).

Within group B, statistically significant differences were found in samples submersed in saliva to which forces of 1 N vs. 10 N (*p* = 0.048) were applied. As for group D, statistically significant differences were recorded in samples submersed in cola solution after applying forces of 1 N vs. 5 N (*p* = 0.021) and 1 N vs. 10 N (*p* = 0.036).

**Figure 5:**
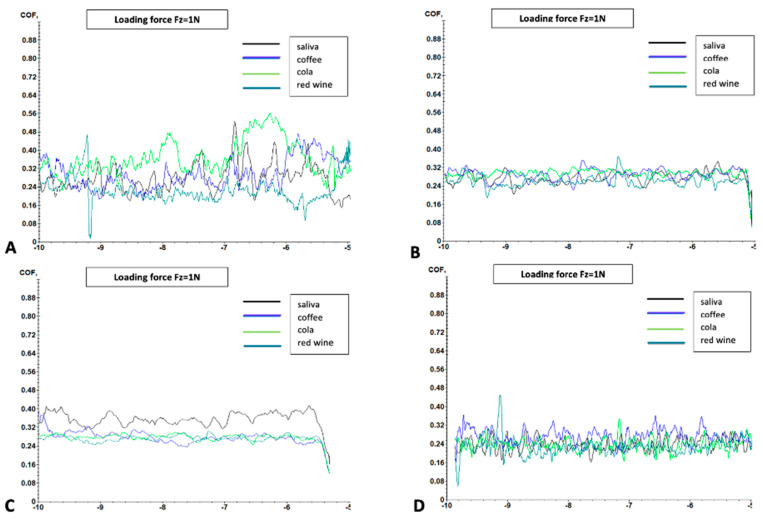
Variation of the coefficient of friction (COF) with the scratch distance (Y) of the samples in each group after submersion in each of the 4 solutions. (**A**)—Samples made of resin-based sealant; (**B**)—samples made of heavy flow resin; (**C**)—samples made of flowable resin; (**D**)—samples made of conventional nanohybrid composite resin.

Figure 5 shows the scratching behavior defined by the variation of the coefficient of friction of a sample from each material submersed in each of the 4 tested solutions.

## 4. Discussion

In the present study, the null hypothesis was rejected. Our findings clearly demonstrated significant decreases in mechanical properties, such as Vickers microhardness, Young’s modulus of elasticity and scratch resistance, for all tested materials following submersion in coffee, cola and red wine solutions. This degradation can be attributed to the acidic environment’s impact on the organic matrix and filler interactions [15]. The acids facilitated the hydrolysis of the polymer matrix and weakened the bonds between filler particles and the organic matrix [16], as evidenced by the greater reductions in properties of materials with lower filler content and more hydrophilic matrices. The obtained results showed consistent and statistically significant deviations from the expected outcomes under null conditions.

In the present study we evaluated the mechanical properties of four resin-based dental restorative materials by using different tests. Thus, Vickers surface microhardness testing, Young’s modulus of elasticity testing, and scratch resistance analysis were performed. These tests had the advantage of being simple to perform, repeatable and not significantly damaging the study samples [17]. Material microhardness (in this study Vickers microhardness) has been defined as the resistance of the material to a vertical pressure exerted by an indenter when the material is under static conditions, while scratch resistance was defined as the resistance of the material to dynamic deformation of an indenter [18,19]. These experiments were necessary to test the strength and the durability of dental composite resins.

The tested materials were chosen from the same manufacturer in order to have similar or almost similar chemical compositions of the organic matrix. This was based on BisGMA, BisEMA and TEGDMA monomers in the nanohybrid flow, heavy flow, and conventional composites and on BisGMA, TEGDMA and BTH monomers in the resin sealant. Studies conducted on restorative materials over the years have shown that each element of the composite resins (organic matrix, filler particles and resin matrix/filler interface) influences the physical, chemical and mechanical properties of the material and can lead to a faster or slower degradation [20].

In some previous studies, it was reported that composite resins with large filler particles have higher wear resistance and microhardness [15,21], while others concluded that composite resins with small and regular filler particles presented significantly increased mechanical properties [22,23].

However, in our study it is not possible to draw conclusions on the importance of filler particle size given that all the tested materials were composed of nanosized particles. Following the analysis of the scratch resistance, more specifically the COF coefficient of friction, the highest value was recorded by the conventional nanohybrid composite nanohybrid resin, followed by the heavy-flow nanohybrid resin, the flow nanohybrid resin and finally the resin-based sealant. A possible explanation of this behavior can be related to the materials’ filler loading. The same order of values was recorded in the Vickers microhardness analysis while in the modulus of elasticity analysis, the conventional nanohybrid composite resin showed the highest mean values, followed by the nanohybrid flow resin, nanohybrid heavy-flow resin and resin sealant.

The durability and wear resistance of composite resins has been correlated in in vitro studies with Vickers microhardness, which evaluates indentation resistance under functional stresses [24]. Previous studies have demonstrated higher Vickers microhardness of the composite resins having nanometer fillers when compared to larger-size ones [25].

Along with the complex conditions of the oral cavity, dental materials are also prone to a continuous degradation process due to contact with food and beverages with different temperatures or pH values [17,26,27].

Young’s modulus of elasticity represents the relative stiffness of a material and the higher the value, the more the material will be able to resist the deformations induced by occlusal stresses [17,28]. In our study, conventional nanohybrid composite resin presented the highest values of Young’s modulus of elasticity after 1–7 and 14 days of evaluation when compared to the other tested materials. In descending order, it was followed by the flowable composite resin, the heavy-flow composite and resin-based sealant. These results could be associated with the results obtained by Vickers microhardness or scratch resistance analysis. For all the tested materials, the microhardness, Young’s modulus of elasticity and scratch resistance values decreased after 14 days of submersion in the acidic beverages. These results are confirmed by other authors [16,17,20,23,24].

For the resin-based sealant, the Young’s modulus values after being submersed for 14 days in red wine solution decreased. These results are partially confirmed by those obtained for the Vickers microhardness analysis where the average values decreased after submersion for 7 days in wine solution. The scratch analysis results show a significant decrease in the mean after submersion for 14 days in cola, coffee and red wine solutions. The behavior of the resin sealant can be explained by the reduced percentage of inorganic filler and, consequently, the better representation of the organic matrix, which has the ability to absorb fluids [17].

The results obtained from the analysis of Vickers microhardness and scratch resistance agree with the results of other studies that showed similar values, and they are also in accordance with the percentage of inorganic filler [17,29]. For Young’s modulus, flowable resins obtained higher mean values compared to heavy flow resins, and this may be attributed to the small difference in the percentages of inorganic filler, or it may be a consequence of the more homogeneous distribution of particles in one of the two materials [17]. However, for conventional composite resins, the results of Vickers microhardness are in agreement with those obtained for Young’s modulus of elasticity, and this conclusion is also supported by the previous study of Masouras [30]. When analyzing the changes of modulus of elasticity of resin-based materials, heavy-flow resin presented decreased values by 9.2% after submersion in coffee solution for 7 days, and flowable resin showed decreased values by 26.73% after storage for 7 days in coffee solution and for resin-based sealant. Some authors have contradictorily concluded in previous studies that simple submersion of composite resins in water can degrade the material by decreasing its elasticity [18,31] or by keeping it unchanged [32].

For Vickers microhardness analysis, the results obtained in the study showed that for the heavy-flow composite resin, 14 days of submersion in cola solution decreased the microhardness values by 19.2%, 14 days of storage in coffee decreased the values by 30.8% and 14 days of submersion in wine decreased the microhardness by 17.5%. For flowable resin, submersion in cola for 7 days decreased microhardness by 1.7% while storage for 14 days in coffee decreased the values by 29.1%. These results agree with the findings of other studies that found a decrease in microhardness values after submersion in different acidic solutions [24,25,27,29].

When analyzing the scratch resistance, soaking the heavy-flow resin for 14 days in coffee decreased the resistances by 42.8 and storing the conventional composite resin for 14 days in cola-like beverage decreased the values by 59.4%. For the resin-based sealant, the scratch resistance values decreased after 14 days of submersion in each of the three acidic solutions with percentages ranging from 80 to 90%. These results are in line with those obtained by other authors that found a significant decrease of scratch resistance of resin-based materials after submersion in various acidic solutions [17,25].

The cola-like beverage affected the resin restorative materials due to the presence of phosphoric acid which has the ability to soften Bis-GMA monomers [33,34]. Under the conditions of wear resistance, the composites exhibit a defined behavior by delamination, which represents a flaking of the fillers and the occurrence of cracks in the depth of the material [17]. Thus, the results of our study analyzing the scratch resistance are in agreement with other studies’ results, which were based on the higher percentage of filler particles and consequently a more extensive delamination phenomenon [35].

The behavior of a restorative material is dependent on several factors and components, not only on the filler particles [29]. Of these properties, an important one is the homogeneity of the material so that neither mechanical factors are able to deform the material nor acids come into contact with the resin matrix [26,36]. In the present study, the nanohybrid composites showed a good resistance to degradation, which can be attributed to the high filler particle density that reduces permeability and hydrolysis effects at the resin–filler interface. This observation is supported by the literature, which emphasizes that uniform distribution of small-sized fillers may increase the microhardness and wear resistance of composites [25,27]. On the other hand, the organic matrix, consisting mainly of hydrophilic monomers, such as Bis-GMA and TEGDMA, is more susceptible to water absorption and plasticization under the influence of acids, mechanisms which degrade the mechanical properties by reducing intermolecular bonds and decreasing rigidity [34]. For example, coffee and acidic beverages, through their low pH, caused a significant decrease in the microhardness and elasticity of the materials, which is an effect observed particularly in materials with low filler content. This underlines the importance of an optimal combination of filler composition and the chemical nature of the matrix to minimize the effects of degradation under clinical conditions.

According to research conducted by Guler et al., the typical individual drinks an average of 3.2 cups of coffee daily. Considering that a single cup is usually consumed over 15–20 min, submersion of the samples continuously for 14 days is equivalent to simulating coffee exposure over the course of approximately one year [37].

From the results obtained from each of the three tests, we observed that the material with the highest increased strength is the conventional nanohybrid composite resin, followed by the heavy-flow nanohybrid resin, the flowable nanohybrid resin and the resin sealant. The results can be explained by the filler loading and by the type of the polymer matrix [16]. Thus, monomers can be more or less hydrophilic, with this property indicating higher or lower water sorption. The acidic solutions in which the materials have been soaked can act by using several mechanisms, such as diffusion in the polymer network, in which water molecules fill the empty spaces between the polymer chains and plasticize the organic matrix, or by hydrolysis reaction, in which the siloxane bonds are degraded [38]. Unless proper finishing and polishing is not performed, the surface of the material becomes roughened by acid attack, thus wear resistance, scratch resistance and elasticity will be considerably reduced [39].

The limitation of this in vitro study is the impossibility of reproducing all the characteristics of the oral environment where both chemical, physical and mechanical factors act on restorative materials in a continuous, uninterrupted manner. Future studies to investigate the mechanical and tribological properties of dental resin-based materials could focus on increasing the variety of acidic solutions to test the variability of the oral environment and eating habits. Furthermore, prolonged immersion and cyclic thermal or mechanical stresses may mimic more realistic aging conditions for restorative materials. Inclusion of newer composite resin alternatives, with varied filler compositions or innovative chemical recipes of the organic components, could help explore improvements in durability and resistance to acid degradation. Finally, further in vivo or clinical studies are recommended to validate laboratory results and evaluate the performance of the materials under real oral conditions.

## 5. Conclusions

The submersion of conventional nanohybrid, flowable, heavy-flow composite resins and resin-based sealants in coffee, cola and red wine solutions changes their mechanical properties (Young’s modulus of elasticity, Vickers microhardness and scratch resistance).

The most resistant resin-based material to acid attack was the conventional nanohybrid composite resin, followed by heavy-flow resin, flowable resin and resin-based sealant.

Fourteen days of day submersion in coffee, Pepsi cola and red wine beverages of the resin-based restorative materials tested decreased mechanical properties of resin-based restorative materials.

Therefore, identifying the optimal dental restorative materials can enhance the longevity of restorations and support the advancement of personalized dental care for patients.

## Figures and Tables

**Table 1:** Tested materials: name, manufacturer and composition.

Materials	Organic Matrix	Fillers	Group Distribution
Grandio SO Flow (VOCO)	BisGMA BisEMA TEGDMA	Ceramic glass filler with an average particle size of 1 µm; functionalized silicon dioxide nanoparticles (mineral particles 81% by weight)	Group A (*n* = 40)
Grandio SO Heavy Flow (VOCO)	BisGMA BisEMA TEGDMA	Ceramic glass filler with an average particle size of 1 µm; functionalized silicon dioxide nanoparticles (mineral particles 83% by weight)	Group B (*n* = 40)
Grandio Seal (VOCO)	BisGMA TEGDMA BTH	Ceramic glass filler with an average particle size of 1 µm; functionalized silicon dioxide nanoparticles (mineral particles 70.2% by weight)	Group C (*n* = 40)
Grandio SO (VOCO)	BisGMA BisEMA TEGDMA	Ceramic glass filler with an average particle size of 1 µm; functionalized silicon dioxide nanoparticles 20–40 nm in size (mineral particles 83% by weight; 73% by volume); pigments (titanium dioxide, metal oxide).	Group D (*n* = 40)

Abbreviations: BisGMA: Bisphenol A diglycidyl ether dimethacrylate; TEGDMA: tri-ethylene glycol dimethacrylate; BisEMA: Bisphenol A polyethylene glycol diethyl ether dimethacrylate; BTH:2,6-di-t-butyl-4-methyl phenol.

## Data Availability

All the data presented in this study are available within the article.
